# The novel role of Yin Yang 1 in acute rejection of liver allografts through activation of dendritic cells

**DOI:** 10.3389/fimmu.2025.1597779

**Published:** 2025-05-19

**Authors:** Yi Chen, Jie Wang, Liping Hong, Hongtao Wang, Wubing He, Lihong Chen

**Affiliations:** ^1^ Department of Pathology and Institute of Oncology, The School of Basic Medical Sciences, Fujian Medical University, Fuzhou, Fujian, China; ^2^ Department of Pathology, Mengchao Hepatobiliary Hospital of Fujian Medical University, Fuzhou, Fujian, China; ^3^ Diagnostic Pathology Center, Fujian Medical University, Fuzhou, Fujian, China; ^4^ Provincial Clinical Medical College, Fujian Provincial Hospital, Fujian Medical University, Fuzhou, Fujian, China

**Keywords:** Yin Yang 1, acute rejection, dendritic cells, liver transplantation, inflammatory pathways

## Abstract

**Introduction:**

Acute rejection is a critical complication after liver transplantation, contributing significantly to transplant dysfunction and recipient mortality. Yin Yang 1 (YY1), a zinc-finger transcription factor, has an undefined role in liver allograft acute rejection, despite its broad expression and regulatory potential in immune responses.

**Methods:**

To investigate YY1's role, we used an MHC Class II-mismatched rat liver transplantation model. Allografts were harvested on post-transplant days 5 and 10 for YY1 expression analysis in inflammatory cells around recipient liver central veins. *In vitro*, dendritic cells (DCs) were transfected to overexpress YY1, and their surface markers (CD80, CD86, MHC II) and cytokine production (TNF-α, IL-6) were assessed. Naïve CD4^+^ T cells were co-cultured with YY1-overexpressing DCs to evaluate their polarization towards inflammatory phenotypes (IL-17, IFN-γ production).

**Results:**

YY1 expression was elevated in inflammatory cells of allografts on days 5 and 10 post-transplant, correlating with increased serum transaminases and inflammatory cytokines. YY1-overexpressing DCs showed heightened expression of CD80, CD86, and MHC II, along with augmented TNF-α and IL-6 production. These YY1-activated DCs drove naïve CD4^+^ T cells to produce higher levels of IL-17 and IFN-γ, indicating polarization towards a proinflammatory Th17/Th1 phenotype.

**Discussion:**

YY1 promotes DC activation and naïve T cell polarization towards inflammatory phenotypes, thereby contributing to acute rejection in liver transplantation. Targeting YY1 may offer a therapeutic strategy to mitigate acute rejection and improve transplant outcomes. Further research is warranted to explore YY1's regulatory mechanisms and therapeutic potential in liver transplantation.

## Introduction

Liver transplantation is the ultimate treatment for end-stage liver disease, and successful liver transplantation significantly prolongs patient survival (1). Compared with other organ transplants, such as kidney and lung transplantation, liver transplantation generally exhibits milder immune rejection (2). However, acute rejection still occurs in some patients after liver transplantation, severely reducing the survival rate of the donor liver. As a result, lifelong systemic administration of immunosuppressive drugs is often required to ensure long-term survival of the transplanted liver, although these drugs may cause malignant tumors or infections due to the immunosuppressed state (3). Therefore, elucidating the mechanisms underlying immune rejection and immune tolerance after liver transplantation is of great significance for the diagnosis and development of new therapeutic strategies for acute rejection.

Abnormal activation of various immune cells is involved in the mechanism of immune rejection in liver transplantation. Among these, both donor-derived and recipient dendritic cells (DCs) have long been recognized as important factors in the direct, indirect, and semi-indirect pathways of antigen presentation that trigger acute liver allograft rejection (4, 5). Compared with other non-lymphoid organs, a higher number of interstitial DCs reside in the portal tracts and around hepatic veins, with some cells scattered interstitially between hepatocytes (6). Consequently, a small number of donor-derived DCs are passively transferred to the recipient as passenger cells after liver transplantation. In the direct pathway of antigen presentation during acute rejection, these DCs can migrate to the recipient’s lymph nodes and present alloantigens on their MHC class I and II molecules to recipient naïve T cells. The indirect pathway involves recipient DCs capturing and processing alloantigens before presenting them to naïve T cells (7). In the semi-indirect pathway, recipient DCs acquire non-self MHC molecules via cell-to-cell contact or fusion of exosomes, subsequently activating naïve T cells. Although less potent than the direct pathway, the indirect and semi-indirect pathways are not only capable of initiating acute rejection but also contribute to subsequent episodes of rejection (8). The common outcome of these three pathways is the accumulation of mature DCs, followed by the differentiation of naïve T cells into Th1 and Th17 subgroups. Th1 cells secrete IL-1, IFN-γ, and TNF-α upon activation and play an important role in immune regulation, autoimmune disease, and transplant immune rejection. Studies have confirmed that Th17 cells are involved in acute immune rejection of kidney, lung, heart, and other transplants through the release of inflammatory cytokines such as IL-17. The cross-talk between DCs and naïve T cells during liver transplantation is associated with the abnormal expression of damage-associated molecular pattern molecules, transcription factors encoding inflammatory mediators, and other factors, which may serve as therapeutic targets and early warning indicators for the prevention of liver allograft rejection.

Yin Yang 1 (YY1), a member of the zinc-finger class of DNA-binding proteins and belonging to the GLI-Kruppel family, is an evolutionarily highly conserved transcription factor. The N-terminal region of YY1 (amino acids 1-100) contains a transcriptional activation domain and is involved in electrostatic interactions with positively charged proteins and nuclear localization (9, 10). In contrast, the central region and the sequence near the carboxyl-terminal region of YY1 are responsible for transcriptional repression (11). Furthermore, the C-terminal region of YY1 mediates DNA binding via four C_2_H_2_-type zinc-finger motifs, which allow YY1 to interact with and recruit a diverse array of other transcription factors and various transcriptional regulators (12, 13). Therefore, YY1 can function as either a transcriptional activator or repressor in many different cellular processes, depending on the chromatin context and environment, as suggested by its name. Previous studies have shown that several T cell cytokine promoter regions, including those of IL-4 (14), IL-5, and IFN-γ (15), contain consensus YY1 binding sites. Thus, YY1 may play an important role in inflammation-related diseases. In rheumatoid arthritis, YY1 has been found to promote pathogenic Th17 cell differentiation by interacting with T-bet and participating in the pathophysiological process (16). Other research has reported that partial YY1 deficiency can suppress the expression of IL-4 and IFN-γ and attenuate the differentiation of naïve T cells toward the Th2 phenotype (17). Although these studies have emphasized the key role of YY1 in T cell differentiation and its ability to regulate inflammatory factor expression, no research has yet revealed whether YY1 is also involved in the maturation of DCs or the cross-talk between DCs and naïve T cells. Given that Th1- and Th17-dominated immune responses are observed in acute rejection after liver transplantation, elucidating the function of YY1 in these processes may help explain the mechanisms underlying the polarization of the Th1/Th2 paradigm toward Th1 and the accumulation of Th17 cells, which are responsible for immune-mediated injuries after liver transplantation.

In this study, we established a rat liver transplantation model and observed that increased expression of YY1 was associated with the accumulation of inflammatory cells in allogeneic transplanted livers. *In vitro* studies demonstrated that overexpression of YY1 could activate bone marrow-derived mouse DCs and consequently promote the differentiation of CD4^+^ T cells into Th17 and Th1 subsets. These findings suggest that YY1 may play an important role in the acute rejection of liver transplantation by modulating the immune response.

## Materials and methods

### Animals

Specific pathogen-free male Lewis rats and Brown Norway (BN) rats, aged 6–8 weeks and weighing 250–300 g, were used in this study. Animals were purchased from the Laboratory Animal Center of Fujian Medical University (Fujian, China) and maintained under specific pathogen-free conditions with a constant temperature (20 ± 2°C) and humidity (50 ± 10%). All experimental procedures were approved by the Ethics Committee of Fujian Medical University (No. 2015-29) and conducted in accordance with the NIH Guidelines for the Care and Use of Laboratory Animals.

### Orthotopic liver transplantation

Orthotopic liver transplantation was performed using the modified two-cuff technique as previously described (17, 18). In the allogeneic group, livers from 14 Lewis rats were orthotopically transplanted into BN rats of similar weight and age. In the syngeneic group, livers from 14 pairs of Lewis rats were transplanted between donors and recipients of the same strain. As a control, 14 Lewis rats underwent a sham operation, in which only the abdominal cavity was opened and closed under anesthesia for 35 minutes to simulate the procedure and duration of liver transplantation. Briefly, rats in the syngeneic group and allogeneic group were administered an intraperitoneal injection of 2% (w/v) sodium pentobarbital solution (0.3 ml per 100 g body weight; Eskonarkon, Switzerland). During the induction of deep anesthesia, vital signs of the rats (such as respiration and heartbeat) were monitored in real time to ensure animal safety. After the abdominal cavity was opened, the hepatic artery, portal vein, and inferior vena cava were fully exposed. The surrounding tissues of the liver were dissected, and cuffs were placed on the hepatic artery and portal vein to ensure stable anastomosis. Subsequently, the blood vessels and bile duct were severed, and the liver was completely removed and placed in 4°C cold physiological saline for later use. Meanwhile, recipient BN rats were anesthetized and a midline abdominal incision (3 cm) was made to expose the recipient liver. The hepatic artery, portal vein, and hepatic vein were ligated stepwise, and the bile duct was severed to completely remove the recipient liver, avoiding damage to other organs and blood vessels. The hepatic artery, portal vein, and hepatic vein of the donor and recipient were sequentially anastomosed using the cuff technique. The vascular clamps were released to restore blood flow, and the blood supply to the transplanted liver was observed to ensure it was adequate. The bile ducts were connected using 8–0 sutures to prevent bile leakage. Finally, the abdominal cavity was irrigated with physiological saline containing antibiotics, followed by closure of the abdominal wall in layers. Postoperatively, enrofloxacin (10 mg/kg) was administered via intramuscular injection once daily for two consecutive days to prevent infection. Additionally, carprofen (5 mg/kg) was administered subcutaneously twice daily for three consecutive days to alleviate pain. On postoperative days 5 and 10, three rats from each group were euthanized by overdose anesthesia to obtain transplanted liver tissues and peripheral blood for further analysis.

### Assessment of acute rejection in liver transplantation model

To assess the successful establishment of the liver transplantation and acute rejection models, we measured the levels of alanine aminotransferase (ALT) and aspartate aminotransferase (AST) in the serum and performed hematoxylin and eosin (H&E) staining on the transplanted liver tissues. The criteria for successful establishment of the acute rejection model included (1): elevated levels of ALT and AST, and (2) histological evidence of extensive infiltration of inflammatory cells, vascular lesions, and damage to biliary epithelial cells in the livers of the acute rejection group. The grafted liver tissues were fixed in paraformaldehyde, embedded in paraffin, and serially sectioned into 4-µm thick slides. Representative H&E staining results were used to evaluate the degree of acute rejection. At 5 and 10 days after transplantation, blood samples were collected from the caudal vein of recipient rats under ether anesthesia and centrifuged at 5000 × g for 15 minutes to separate the serum. The serum levels of AST and ALT were measured using commercial kits (C010-2, C009-2, E003-2, Nanjing Jiancheng, Nanjing, China) according to the manufacturer’s instructions.

### Immunohistochemistry

For immunohistochemistry, the slides were deparaffinized, and antigen retrieval was performed using sodium citrate-EDTA antigen repair solution under high pressure. Endogenous peroxidase activity was blocked with 3% H_2_O_2_. The slides were then incubated with rabbit monoclonal anti-CD3 antibody (1:500; ab86883, Abcam, USA) or monoclonal anti-CD86 antibody (1:200; ab239075, Abcam) overnight at 4°C. Subsequently, the sections were stained using the Streptavidin/Peroxidase Histostain™ Plus Kit (ZSGQ-BIO, Beijing, China) according to the manufacturer’s instructions. The number of positive cells per field was analyzed and calculated using Image-Pro Plus software version 6.0 (Media Cybernetics, USA).

### Western blotting analysis

Grafted liver tissues were collected from all groups and subjected to total protein extraction using RIPA Lysis and Extraction Buffer (Thermo, USA). Protein concentrations were quantified using a BCA kit (23250, Thermo, USA). Subsequently, 60 µg of protein was separated by 12% sodium dodecyl sulfate polyacrylamide gel electrophoresis (SDS-PAGE) and transferred to nitrocellulose membranes. The membranes were blocked with 5% skim milk and incubated with primary antibodies against YY1 (1:1000, ab245365, Abcam) and β-actin (1:2000, ab8226, Abcam) overnight at 4°C. After washing with PBS, the membranes were incubated with peroxidase-conjugated secondary antibodies. The signals were visualized using enhanced chemiluminescence (Pierce, Rockford, IL, USA) according to the manufacturer’s instructions. The intensity of the bands was semi-quantitatively analyzed using ImageJ software (NIH, USA) to evaluate the expression levels of the target proteins.

### RNA extraction and quantitative real-time PCR

Grafted liver tissues were ground into tissue homogenates, and total RNA was extracted using TRIzol reagent (Invitrogen, Carlsbad, CA, USA). Subsequently, total first-strand cDNA was synthesized using the PrimeScript 1st Strand cDNA Synthesis Kit (Takara, Japan). RT-qPCR reactions were then performed using the TransStart Top Green qPCR SuperMix (TransGen, Beijing, China). The primers for analyzing the YY1 gene were designed as follows: 5’-GAGTCCACGTCTGTGCAGAAT-3’ (forward) and 5’-CGTCGAAGGGGCACATAG-3’ (reverse). The primers for 18S rRNA (used as an internal control) were 5’-AGAAACGGCTACCACATCC-3’ (forward) and 5’-CACCAGACTTGCCCTCCA-3’ (reverse). The cycle threshold (Ct) values were recorded, and the relative expression levels of the YY1 gene were analyzed using the 2−ΔΔCt method after normalization to 18S rRNA.

### Tandem mass tag-based proteomic analysis

The frozen grafted liver tissues were lysed in RIPA lysis buffer (Beyotime Biotechnology, Beijing, China) and homogenized using a tissue grinder. The lysates were then sonicated using a high-intensity ultrasonic processor. After centrifugation at 12,000 × g for 20 minutes, the supernatants were collected and transferred into Pierce™ Top 12 Abundant Protein Depletion Spin Columns (Thermo Fisher Scientific, Waltham, MA) to remove high-abundance proteins. The protein concentration in the supernatants was quantified using the BCA Protein Assay Kit (Thermo Fisher Scientific). A total of 200 µg of protein from each sample was reduced by incubation with 5 mM dithiothreitol (DTT) at 56°C for 30 minutes, followed by alkylation with 11 mM iodoacetamide in the dark for 15 minutes. The samples were then washed twice with 8 M urea (pH 8.5) and centrifuged at 12,000 × g for 20 minutes. The supernatants were replaced with TEAB solution for protein digestion. After overnight digestion with trypsin, 100 µg of the resulting peptides was labeled using the TMT10-plex™ Isobaric Label Reagent Set (Thermo Fisher Scientific) according to the manufacturer’s instructions. The labeled peptides were fractionated by high-pH reversed-phase high-performance liquid chromatography (HPLC) using an HPLC system (Shimadzu, Tokyo, Japan). The fractions were then subjected to nanoelectrospray ionization followed by tandem mass spectrometry (MS/MS) on a Q Exactive mass spectrometer (Thermo Fisher Scientific) coupled online to the HPLC. The raw data were processed using Proteome Discoverer 2.2 (Thermo Fisher Scientific). Proteins with fold changes >1.20 or <0.83 and an adjusted significance level (P-value) < 0.05 between two comparable groups were considered differentially expressed proteins (DEPs).

### Separation and cultivation of rat bone marrow-derived dendritic cells

Bone marrow-derived dendritic cells (BMDCs) were generated from Lewis rats according to previously described methods. Briefly, bone marrow cells were isolated by flushing the hind limbs with PBS and filtering through a 40-µm cell strainer. The harvested cells were resuspended and cultured in RPMI-1640 medium containing 10% (v/v) fetal bovine serum (FBS; Life Technologies), 10 mM glutamine, and penicillin/streptomycin. After incubation at 37°C with 5% CO_2_ for 3 hours, non-adherent cells were removed, and the remaining adherent cells were harvested and cultured in RPMI-1640 medium supplemented with 20 ng/ml recombinant GM-CSF and 10 ng/ml IL-4 (Peprotech, USA) for 7 days. The culture medium was refreshed on days 3 and 5 during the cultivation period.

### Establishment of YY1 overexpressed imDCs and surface marker detection

The YY1 gene was PCR-amplified using specific primers (Forward: 5’-AGGTCGACTCTAGAGGATCCCGCCACCATGGCCTCGGGCGACACCCTC-3’; Reverse: 5’-TCCTTGTAGTCCATACCCTGGTTGTTTTTGGCTTTAGCGTG-3’) to verify sequence integrity. The amplification products were resolved on a 1% agarose gel, revealing a 1277 bp band corresponding to the YY1 coding sequence. The target DNA fragments were excised and purified using a Gel Extraction Kit (NA1111, Sigma-Aldrich). Subsequent restriction enzyme digestion with BamHI and AgeI (New England Biolabs) facilitated directional cloning into the pcDNA3.1 mammalian expression vector (Invitrogen). Ligation reactions were performed with T4 DNA Ligase (2011A, Takara Bio) according to the manufacturer’s protocols. Sequence-validated recombinant plasmids (pcDNA3.1-YY1) and empty vector controls were transfected into DCs using Lipofectamine 3000 reagent (L3000015, Thermo Fisher Scientific) under optimized conditions. The experimental groups included: 1) pcDNA3.1-YY1 transfected DCs, 2) empty vector-transfected DCs (negative control), 3) TNF-α-pulsed DCs (positive control), and 4) PBS-treated DCs (baseline control). Forty-eight hours post-transfection, cells were harvested and stained with fluorescence-conjugated antibodies: FITC-anti-CD86 (560957, BD Biosciences), PE-anti-CD80 (557227, BD Biosciences), and FITC-anti-MHC II (563586, BD Biosciences). Surface marker expression was quantitatively analyzed using a FACSCanto II flow cytometer (BD Biosciences) with FlowJo v10.8 software for data processing. Three biological replicates were performed to ensure statistical reliability.

To detect cytokines secreted by DCs, the supernatant from cultured DCs was collected and analyzed using ELISA kits according to the manufacturer’s instructions. The cytokines measured included IFN-γ, IL-6, TNF-α, TGF-β, and IL-10.

### Cytokine profile of T cell response stimulated by YY1 overexpressed DCs *in vivo*

To assess the ability of YY1-overexpressed DCs to influence naïve T cell polarization, DCs transduced with GV492-YY1, DCs transduced with GV492 virus, TNF-α-pulsed DCs, and PBS-pulsed DCs were cultured for 72 hours. Naïve CD4^+^ T cells were isolated from the spleens of healthy Lewis rats using MACS with a mouse CD4^+^ T cell isolation kit (130-100-008, Miltenyi Biotec, Germany). A total of 5×10^4^ DCs were co-incubated with 5×10^5^ naïve CD4^+^ T cells in each well of a round-bottom 96-well plate for 8 hours at 37 °C in the presence of 0.7 µL/mL GolgiStop (BD Biosciences, USA), 50 ng/mL phorbol 12-myristate 13-acetate (PMA) (Sigma, USA), and 750 ng/mL ionomycin (Sigma, USA). After incubation, surface staining was performed with anti-CD4-FITC antibody on ice for 30 minutes. Subsequently, cells were suspended in Fixation/Permeabilization solution (BD Cytofix/Cytoperm kit, BD Biosciences, USA) and intracellular cytokine staining was performed using anti-IFN-γ-APC and anti-IL-17-PE antibodies. Finally, the cell suspensions were analyzed by flow cytometry using a FACSCanto II instrument (BD Biosciences, USA).

### Identification of genes bound by YY1 using ChIP-seq and analysis of downstream pathways

Dendritic cells were transfected with a plasmid overexpressing the transcription factor YY1 (pCMV-YY1) or an empty vector control (pCMV-Null) using electroporation. Forty-eight hours post-transfection, chromatin immunoprecipitation sequencing (ChIP-seq) was performed to identify genome-wide YY1 binding sites. Briefly, cells were cross-linked with formaldehyde, lysed, and chromatin was sonicated to generate fragments of 200–500 bp. Immunoprecipitation was carried out using a validated anti-YY1 antibody (Abcam, ab245365). Subsequently, DNA libraries were prepared using the NEBNext Ultra II DNA Library Prep Kit and sequenced on an Illumina NovaSeq 6000 platform (paired-end, 150 bp). Raw sequencing reads were aligned to the reference genome using Bowtie2, and peaks were called using MACS2 with a significance threshold. Differential YY1-bound regions between YY1-overexpressed and control groups were identified using DESeq2, with a fold-change cutoff of ≥2 and an adjusted p-value <0.05. Target genes associated with differential peaks were annotated using ChIPseeker. Functional enrichment analysis of these genes was performed using the clusterProfiler package in R, incorporating Gene Ontology (GO) terms and Kyoto Encyclopedia of Genes and Genomes (KEGG) pathways, with significance set at a false discovery rate (FDR) <0.1.

### Statistical analysis

Continuous variables are presented as means ± standard deviations (SD). Statistical significance among the three groups was assessed using one-way analysis of variance (ANOVA) followed by Tukey’s *post-hoc* tests. All analyses were performed using GraphPad Prism version 6.0 software (GraphPad Software, San Diego, CA, USA). A *p*-value less than 0.05 was considered statistically significant.

## Results

### Establishment and evaluation of a rat liver transplantation model

**Figure 1:**
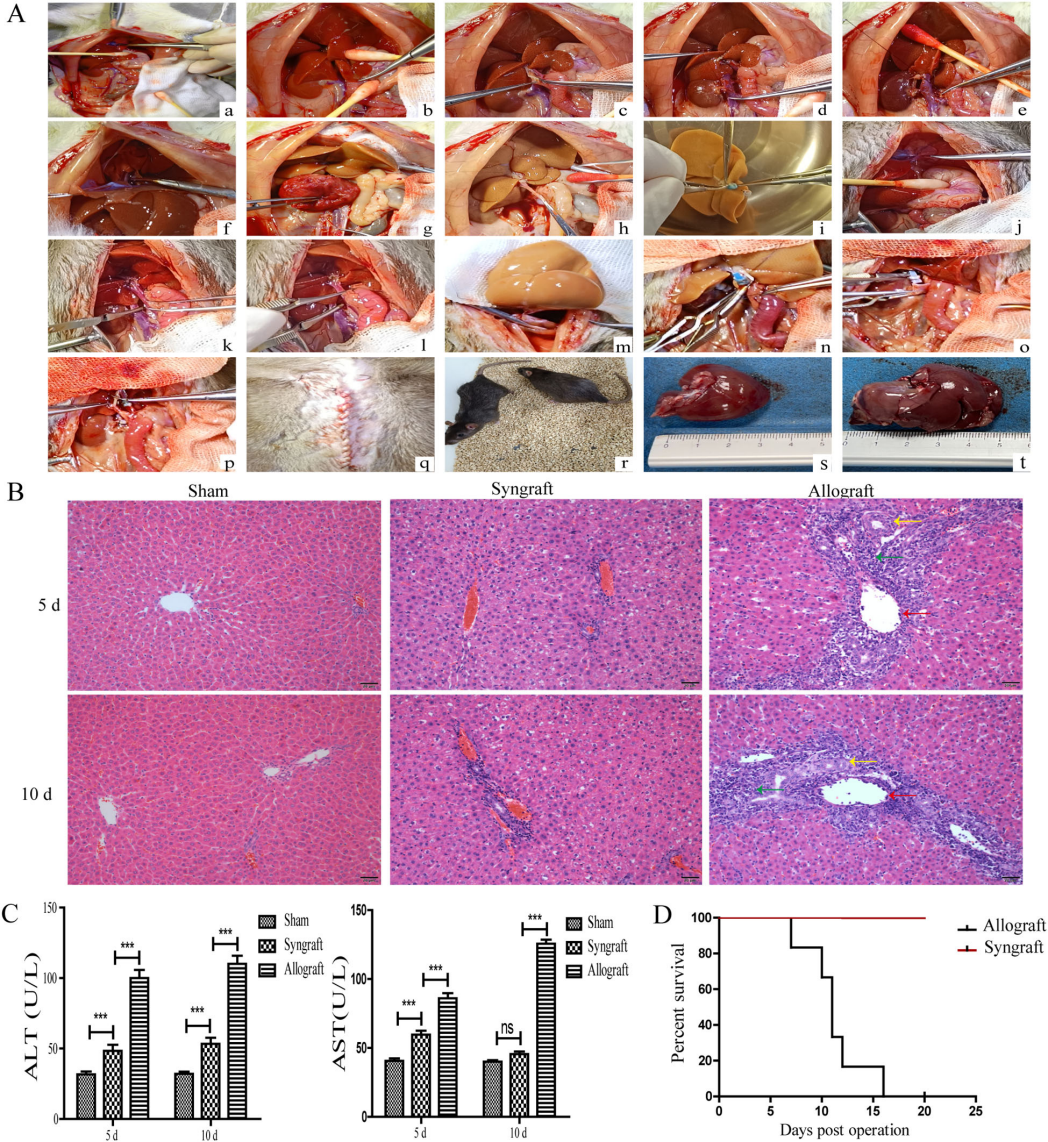
Establishment and evaluation of a rat liver transplantation model. Donated livers from Lewis rats were transplanted into BN rats (allogeneic group) and Lewis rats (syngeneic group). In the sham group, Lewis rats underwent only the opening and closure of the abdomen under anesthesia. **(A)** Schematic illustration of surgical procedures for orthotopic liver transplantation in rats. Donor liver procurement (a-i): (a) Ligation of the left diaphragmatic vein; (b) Mobilization of the caudate and papillary lobes; (c) Placement of the biliary stent; (d) Ligation of the right renal vein; (e) Ligation of the right adrenal vein; (f) Occlusion of the thoracic aorta; (g) Heparinization of the donor; (h) Division of the portal vein; (i) Placement of cuffs on the infrahepatic inferior vena cava and portal vein. **(B)** Liver implantation in the recipient (j–r): (j) Ligation of the left diaphragmatic vein; (k) Occlusion of the infrahepatic inferior vena cava; (l) Occlusion of the portal vein; (m) Suture of the suprahepatic inferior vena cava; (n) Connection of the portal vein cuff; (o) Connection of the infrahepatic inferior vena cava cuff; (p) Connection of the biliary stent; (q) Closure of the abdominal wall; (r) Postoperative recovery. Donated livers in the allogeneic group (s). Donated livers in the syngeneic group (t). **(B)** Histopathology analysis of orthotopic livers from recipient rats (magnification, ×200). Grafted liver tissues were obtained and stained with H&E. Compared with the syngeneic and sham groups, liver tissues in the allogeneic group showed the most severe rejection, characterized by phlebitis (indicated by red arrows), mixed inflammatory cell infiltration (indicated by green arrows), and biliary epithelitis (indicated by yellow arrows). **(C)** Concentration of ALT and AST in the serum of recipient rats. At 5 and 10 days after transplantation, serum samples were collected from recipient rats in all three groups to measure the concentration of ALT and AST. Recipient rats in the allogeneic group had the highest serum levels of ALT and AST. **(D)** Survival curve of Rats After Liver Transplantation. The mice in the allogeneic group died within 15 days after surgery, while no deaths were observed in the syngeneic group. n = 14 in each group. All experiments were performed three times, and data are presented as the mean ± SD. ***p < 0.001; ns, not significant.

After the livers of Lewis rats were orthotopically transplanted into BN or Lewis rats, grafted liver tissues and serum samples were collected from recipient rats for H&E staining and serum aminotransferase determination, respectively. Major surgical procedures for orthotopic liver transplantation in rats were showed in **Figure 1A**. As shown in **Figure 1B**, the grafted liver tissues from the allogeneic group exhibited typical features of acute cellular rejection, including phlebitis (indicated by red arrows), mixed inflammatory cell infiltration (indicated by green arrows), and biliary epithelitis (indicated by yellow arrows). The serum levels of ALT and AST in the allogeneic group were significantly higher compared with the other two groups at 5 and 10 days after transplantation (all *p* < 0.05) (**Figure 1C**). The mice in the allogeneic group died within 15 days after surgery, while no deaths were observed in the syngeneic group (**Figure 1D**) (*p* < 0.001).

### Inflammatory cells infiltration and cytokines in recipient rats

**Figure 2:**
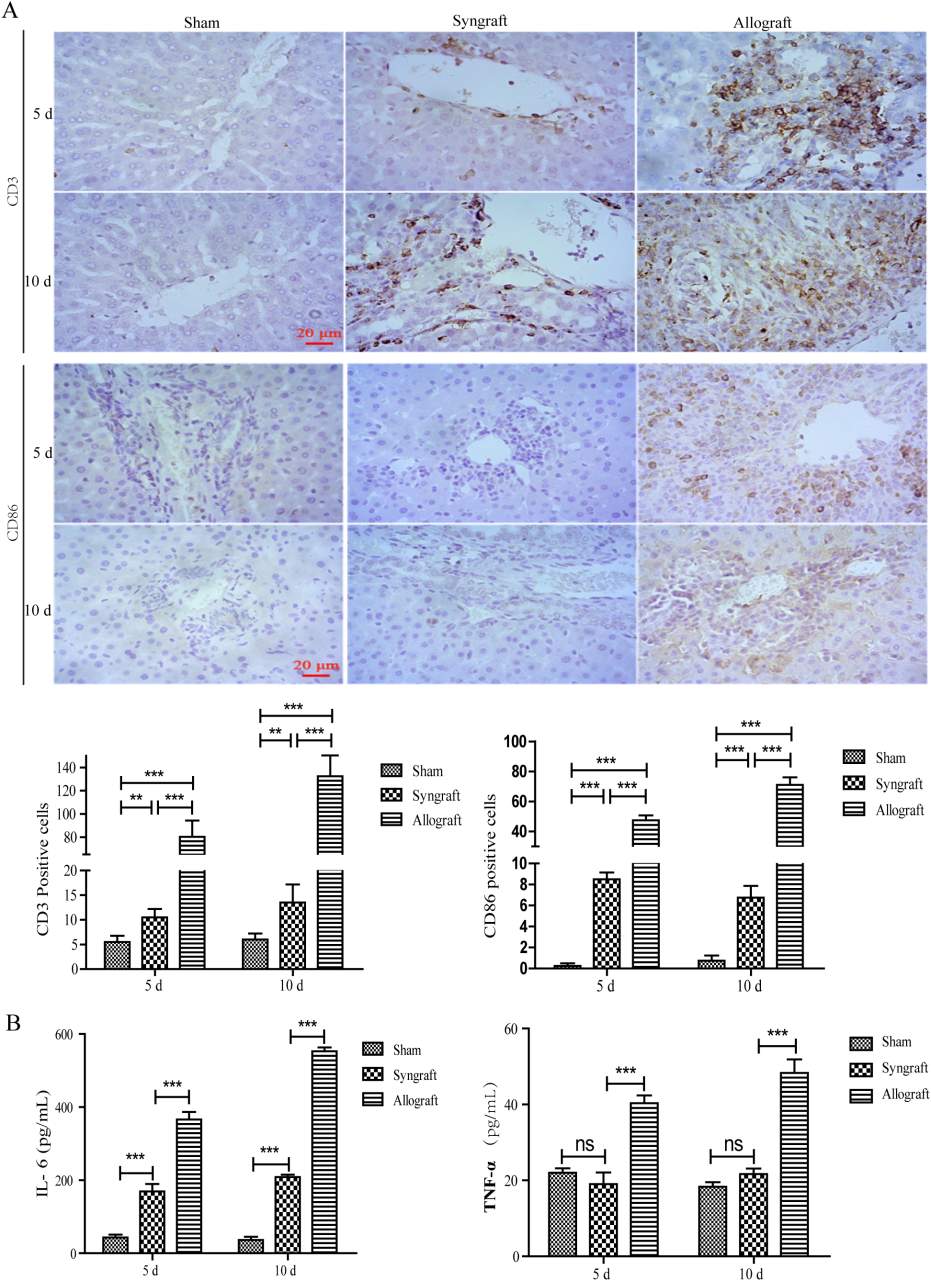
Inflammatory cell infiltration and cytokines in recipient rats. Inflammatory cell infiltration in grafted liver tissues and representative inflammatory cytokines in serum samples were analyzed using immunohistochemistry and ELISA at 5 and 10 days after surgery. **(A)** CD3 and CD86 positive inflammatory cells in liver grafts (magnification, ×200). A significant number of CD3^+^ and CD86^+^ inflammatory cells were observed in the allogeneic group, as confirmed by the statistical analysis presented below. **(B)** Serum concentrations of IL-6 and TNF-α in recipient rats. Serum levels of AST and ALT were highest in the allogeneic group among the three groups at 5 and 10 days after surgery. n = 3 in each group. All experiments were performed three times, and data are presented as the mean ± SD. **p < 0.01, ***p < 0.001; ns, not significant.

To classify the predominant types of inflammatory cells and cytokines, immunohistochemistry and ELISA were performed. Immunohistochemistry revealed that the inflammatory cells in the allogeneic group were primarily composed of CD3^+^ and CD86^+^ cells, indicating that T cells and dendritic cells were the major components (**Figure 2A**). Consistent with the temporal pattern of inflammatory cell infiltration, levels of IL-16 and IFN-γ in the allogeneic group significantly increased at 10 days after transplantation (**Figure 2B**).

### Expression of YY1 Protein and mRNA in the liver tissues of recipient rats

**Figure 3:**
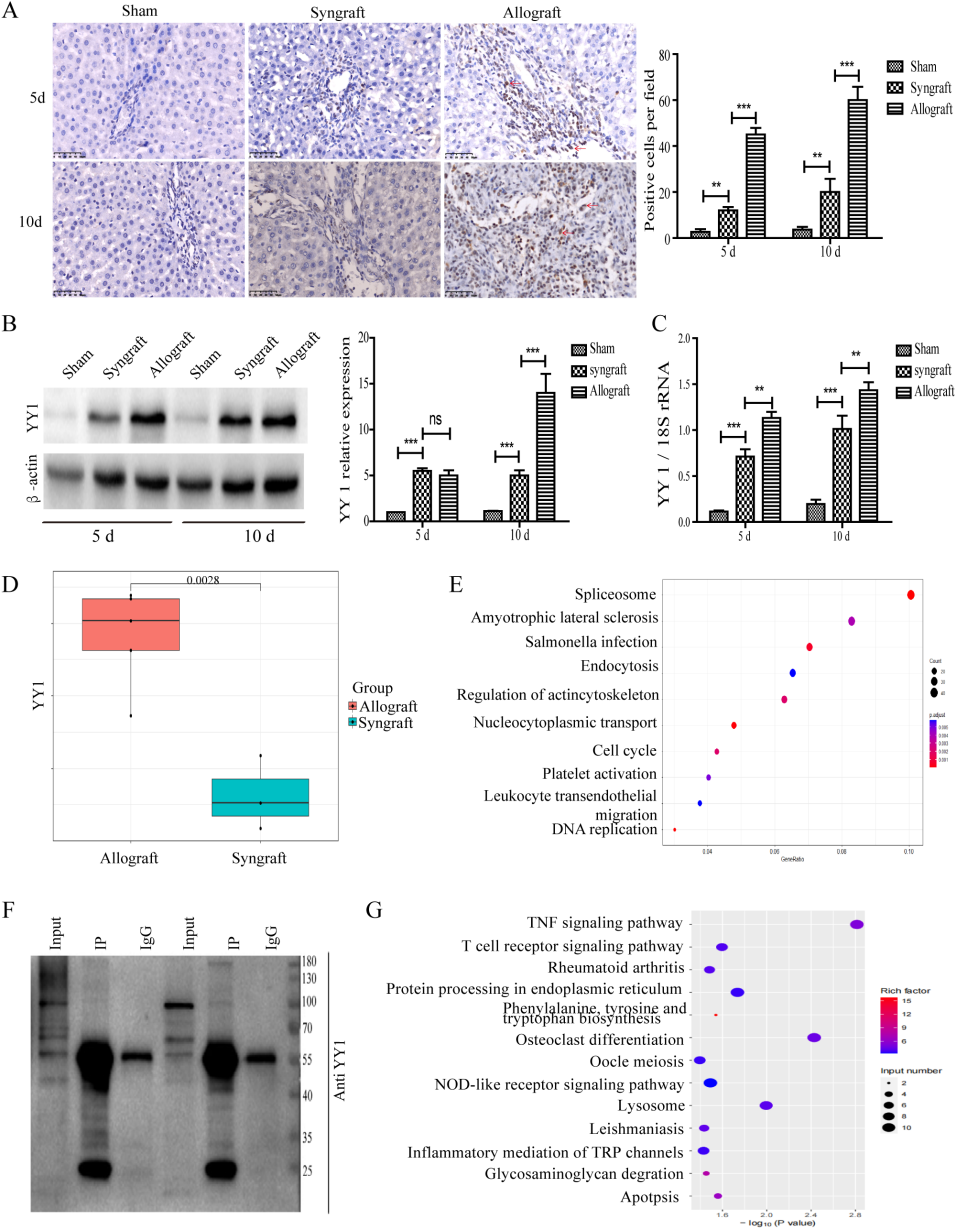
Localization and expression pattern of YY1 protein in liver grafts. Extracted tissues were analyzed using immunohistochemistry, western blot (WB), and qRT-PCR to evaluate YY1 mRNA and protein levels. **(A)** YY1 protein localization by immunohistochemistry (magnification, ×400). YY1 was predominantly localized in the nuclei of infiltrating immune cells in the allograft group, stained brown (indicated by red arrow). **(B, C)** Expression of YY1 protein and mRNA in grafts. YY1 protein and mRNA expression in the allogeneic group increased over time and reached the highest levels among the three groups at 10 days after surgery. n = 3 in each group. The most representative WB results were selected for presentation in the figure. **(D)** YY1 protein expression levels between the allograft and syngeneic groups identified by proteomics. Proteomics analysis showed that YY1 protein expression was higher in the allograft group than in the syngeneic group. **(E)** Further analysis of the proteomics data revealed that genes highly correlated with YY1 can also be enriched in pathways such as lymphocyte migration. **(F)** The representative WB results following the ChIP-seq experiment. The input shows that the target protein is expressed in the sample. Compared with IgG, the target protein is significantly and specifically enriched after IP. **(G)** ChIP-seq results demonstrating that YY1-bound DNA regions are highly enriched in T-cell-related pathways. n = 3 in each group. All experiments were performed three times, and data are presented as the mean ± SD. **p < 0.01, ***p < 0.001; ns, not significant.

To measure the expression of YY1 protein and mRNA in recipient rats, liver graft tissues and serum samples were collected at 5 and 10 days after transplantation. Immunohistochemical analysis revealed that YY1 expression was significantly observed in the nuclei of infiltrated inflammatory cells surrounding the liver portal areas. Moreover, the number of YY1+ cells was markedly higher in the allogeneic group compared to the sham and syngeneic groups (**Figure 3A**). Western blotting analysis showed that YY1 expression gradually increased over time in the allogeneic group (**Figure 3B**), consistent with the trend observed for YY1 mRNA expression (**Figure 3C**). TMT-based proteomic analysis indicated that YY1 protein expression was higher in the allogeneic group than in the syngeneic group, further highlighting the potential role of YY1 in allograft rejection (**Figure 3D**). Further analysis of the proteomics data revealed that genes highly correlated with YY1 can also be enriched in pathways such as lymphocyte migration (**Figure 3E**). Additionally, ChIP-seq results demonstrated that YY1-bound DNA regions were highly enriched in T-cell-related pathways, suggesting that YY1 may regulate inflammation-associated responses (**Figures 3F, G**).

### Overexpression of YY1 triggered the maturation of DC cells and promoted their inflammatory phenotype

**Figure 4:**
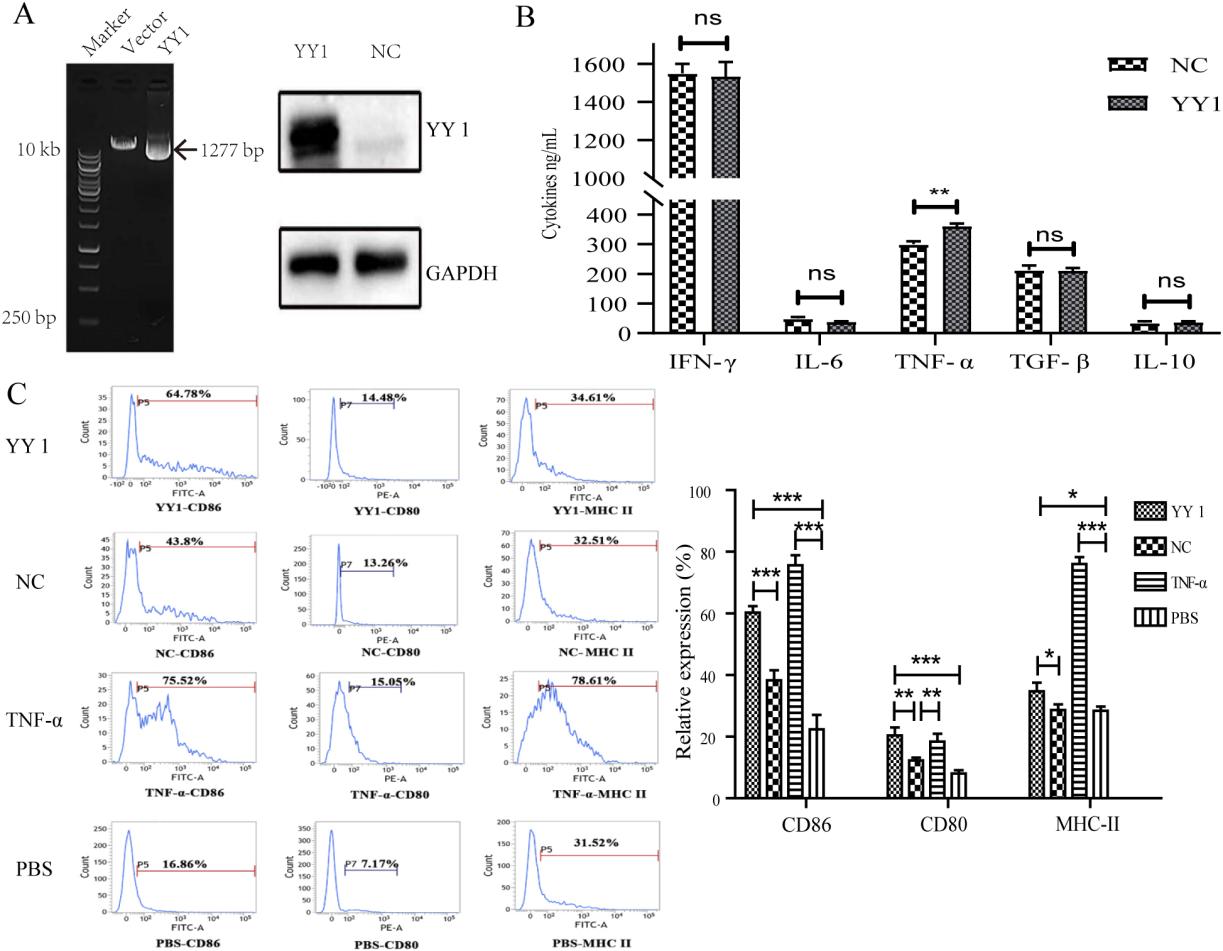
Features and expression of surface markers on YY1 overexpressed DCs. To explore the effect of YY1 overexpression on DC maturation, sequence-validated recombinant plasmids (pcDNA3.1-YY1) and empty vector controls were transfected into DCs. Meanwhile, imDCs were pulsed with PBS (negative control) or TNF-α (positive control). After 8 days of culture, cells were collected for flow cytometry and ELISA assays. **(A)** The YY1 gene was successfully cloned into the pcDNA3.1 vector and expressed in DCs, as confirmed by Western blotting. **(B)** ELISA results showed elevated levels of the inflammatory cytokine TNF-α, indicating an inflammatory phenotype in DCs. **(C)** Flow cytometry revealed increased expression of CD80, CD86, and MHC II in both YY1-overexpressed and TNF-α-pulsed DCs. Statistical analysis of surface marker expression on DCs is shown in the lower panel. All experiments were performed three times, and data are presented as the mean ± SD. *p < 0.05, **p < 0.01, ***p < 0.001; ns, not significant.

Immunohistochemical staining and proteomics analysis have shown that YY1 is upregulated in DCs and infiltrating immune cells. Additionally, ChIP-seq results demonstrated that YY1-bound DNA regions are highly enriched in inflammation-related pathways. To explore the effect of YY1 overexpression on DC maturation, sequence-validated recombinant plasmids (pcDNA3.1-YY1) and empty vector controls were transfected into DCs using Lipofectamine 3000 reagent. The YY1 gene was successfully cloned into the pcDNA3.1 vector and expressed in DCs (**Figure 4A**). ELISA results showed elevated levels of the inflammatory cytokine TNF-α, indicating that DCs exhibited an inflammatory phenotype (**Figure 4B**). Furthermore, FACS analysis revealed increased expression of CD80, CD86, and MHC II in both YY1-overexpressed and TNF-α-pulsed DCs, suggesting that YY1 can trigger DC maturation (**Figure 4C**).

### YY1-overexpressed DCs promoted naïve CD4^+^ T Cells towards an inflammatory phenotype

**Figure 5:**
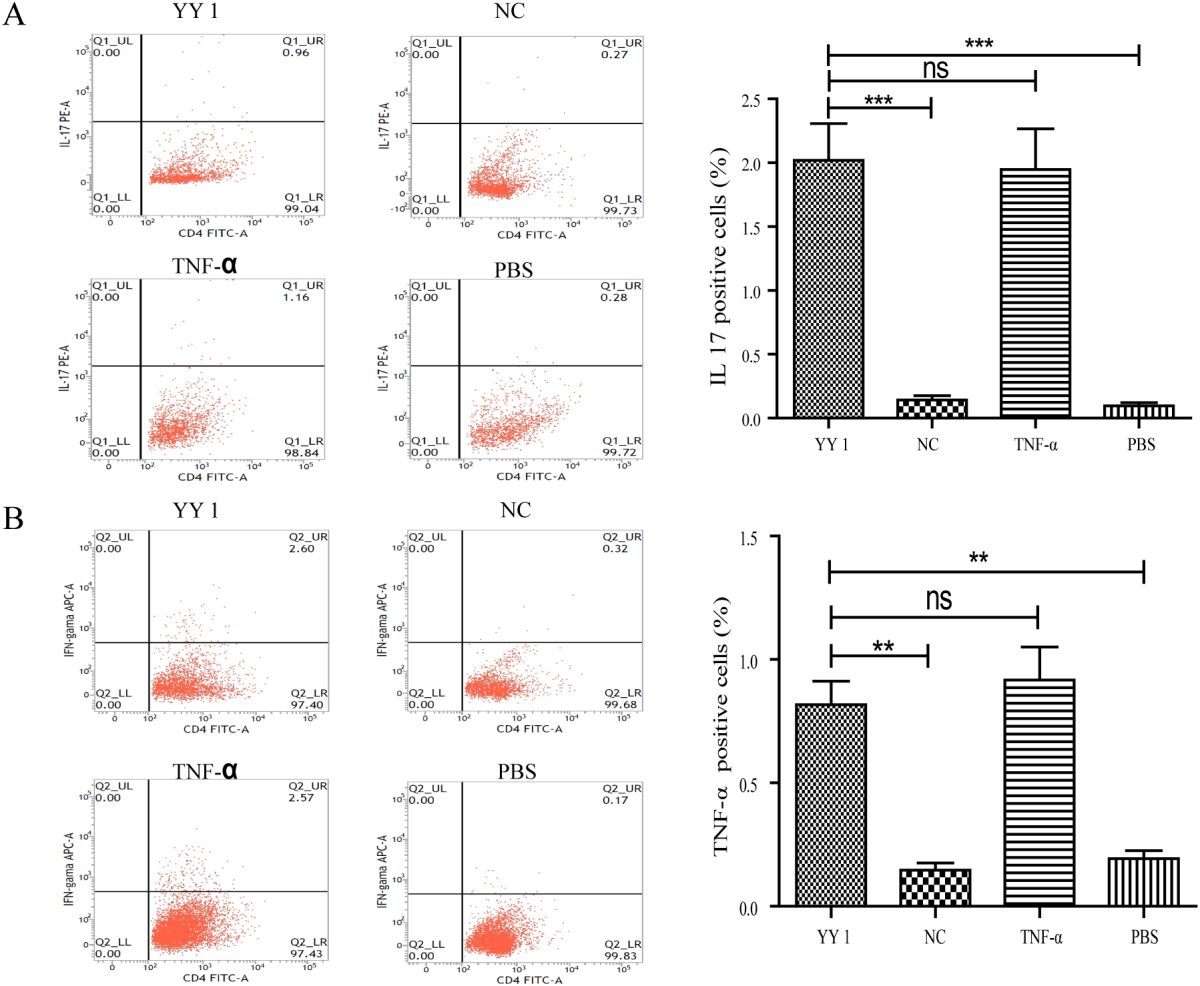
Cytokine secretion of YY1 overexpressed CD4^+^ T cells evaluated by flow cytometry. CD4^+^ T cells were isolated from rat spleens and transfected with lentivirus carrying PCDH-CMV-EGFP-hYY1 or an empty control vector. DCs stimulated with PBS and TNF-α were used as negative and positive controls, respectively. Representative staining and percentages of IL-17 **(A)** and IFN-γ **(B)**-producing CD4^+^ T cells are shown. CD4^+^ T cells pulsed by YY1 overexpressed DCs and those stimulated with TNF-α exhibited higher levels of intracellular cytokines IFN-γ and IL-17 compared to the other two groups. All experiments were performed three times, and data are presented as the mean ± SD. **p < 0.01, ***p < 0.001; ns, not significant.

To assess whether YY1-overexpressed DCs could induce naïve CD4^+^ T cells towards an inflammatory phenotype, DCs from different groups (as previously described) were co-cultured with naïve CD4^+^ T cells isolated from Lewis rats for 72 hours in the presence of Concanavalin A (Con-A). Cytokine profiling revealed that T cells co-cultured with YY1-overexpressed DCs exhibited high levels of the inflammatory cytokines IL-17 and IFN-γ, similar to those co-cultured with TNF-α-pulsed DCs (**Figures 5A, B**).

## Discussion

YY1 is a versatile transcription factor that can act as both an activator and a repressor, regulating the expression of downstream genes in a context-dependent manner. This study investigates the potential role of YY1 in acute rejection following liver transplantation. Using an MHC class II (MHC II)-mismatched rat liver transplantation model, we observed aberrant YY1 expression in inflammatory cells within liver grafts of the allogeneic group. *In vitro* analyses revealed that YY1 overexpression induces DC maturation, which subsequently triggers the differentiation of naïve T cells into Th1 and Th17 subsets.

Rat liver transplantation models are widely used to study transplant rejection mechanisms due to the availability of diverse inbred and transgenic strains with well-defined genetic backgrounds. In this study, we successfully established an acute liver rejection model in rats, characterized by severe portal inflammation, endotheliitis, bile duct destruction, mixed inflammatory cell infiltration, and elevated serum levels of transaminases (ALT/AST) and inflammatory cytokines. Acute liver rejection typically occurs within the first month post-transplantation, with peak incidence at 5–7 days (19). Therefore, we examined pathological changes and YY1 expression at 5 and 10 days post-transplantation. Compared to the syngeneic group, the allogeneic group exhibited dense inflammatory cell infiltration around the central vein of the recipient liver, primarily composed of CD86^+^ DCs and CD3^+^ T cells. YY1 expression was elevated in the nuclei of these infiltrating cells, correlating with the degree of immune cell infiltration. Given that activated donor-derived DCs play a crucial role in T cell function during acute liver rejection, our findings suggest that YY1 may link abnormal immune cell activation to acute liver rejection.

YY1 is a widely expressed zinc-finger DNA-binding transcription factor that interacts with various transcription factors and genes, regulating their activity or expression levels in a pleiotropic manner (20). In inflammation-related diseases such as rheumatoid arthritis (16) and ulcerative colitis (21), YY1 promotes pathophysiological processes by interacting with associated regulators. For instance, YY1 controls Th17 cell pathogenicity in rheumatoid arthritis by binding to the promoter region of the transcription factor T-bet and interacting with T-bet protein (16). Regulatory T cells (Tregs), which are essential for immune homeostasis, are regulated by the transcription factor Foxp3 (22). In a dextran sulfate-induced colitis model, YY1 was shown to reduce Foxp3 expression in Tregs, thereby inhibiting their suppressive function (21). In our study, immunohistochemical staining and proteomics analysis revealed that YY1 was upregulated in DCs and infiltrating immune cells, with YY1-bound genes enriched in endocytosis and leukocyte transendothelial migration pathways. Ischemia-Reperfusion Injury (IRI) refers to the more severe tissue damage that occurs when blood flow is restored (reperfusion) after a period of ischemia in tissues or organs. It is involved in the immune rejection processes of solid organ transplants. Previous studies have found a close relationship between ischemia-reperfusion injury and the expression of YY1 (23). Therefore, we speculate that ischemia-reperfusion injury may promote the expression of YY1, thereby participating in the rejection process of transplanted organs. To further investigate the potential downstream mechanisms following the upregulation of YY1 in DCs, we conducted ChIP-seq experiments. The results showed that genes bound by YY1 can be highly enriched in T cell-related signaling pathways, such as the TNF signaling pathway and the T cell receptor signaling pathway. Meanwhile, proteomics data revealed that genes highly correlated with YY1 can also be enriched in pathways related to lymphocyte migration. These findings suggest that the upregulation of YY1 in DCs may exert its effects by influencing T cell functions.

DCs and T cells are key players in both adaptive and innate immunity and are implicated in inflammation-related diseases, including acute allograft rejection (24). Given the elevated YY1 expression in the nuclei of infiltrating DCs and T cells, we hypothesized that YY1 might activate DCs and promote acute allograft rejection by polarizing naïve T cells towards an inflammatory phenotype. Consistent with this hypothesis, YY1-overexpressed DCs exhibited increased surface expression of CD80, CD86, and MHC II, indicating enhanced maturation. Co-culture experiments showed that YY1-overexpressed DCs induced naïve CD4^+^ T cells to produce high levels of IL-17 and TNF-γ. Th17 cells are pro-inflammatory and can inhibit *Treg* function, thereby altering the balance of immune responses (25, 26). Thus, our data support the notion that YY1 activates DCs, promoting Th17 cell differentiation and subsequent immune-mediated injury. However, the precise molecular mechanisms underlying YY1’s effects on DCs and the upstream regulators controlling YY1 expression remain to be elucidated. Hepatic dendritic cells (HDCs) possess inherent tolerogenic characteristics that contribute to prolonged graft survival, whereas BMDCs typically exhibit immunogenic features (27). In our *in vitro* mechanistic studies, we utilized BMDCs (representing the peripheral DC subset) to investigate the regulatory role of YY1. By overexpressing YY1 in this subset, we revealed that YY1 can also modulate the phenotype of conventional dendritic cells (cDCs), namely BMDCs. Although our *in vitro* experiments did not directly isolate and compare the hepatic DC subsets, our *in vivo* liver transplantation model, as well as existing literature, strongly support the key regulatory function of YY1 in liver-resident DCs (28). In future studies, we will continue to explore and compare the roles of YY1 in isolated hepatic DCs versus peripheral DCs.

This study also has certain limitations. First, all our research was conducted at the rat level, without any related analysis in human tissues. In future studies, we will focus on collecting relevant biopsy materials and validate the conclusions drawn from our animal experiments in human tissues. Additionally, this study did not assess the feasibility of small-molecule inhibitors to antagonize YY1 and its regulatory network. In subsequent research, we will employ high-throughput screening of compounds or protein-molecule docking simulations to identify potential antagonists of YY1, in order to analyze the potential role of targeting YY1 in preventing immune rejection in liver transplantation.

In conclusion, our study demonstrates that YY1 expression is associated with acute liver allograft rejection. YY1 overexpression activates rat bone marrow-derived DCs, characterized by increased expression of CD40, CD86, and MHC II. These mature DCs polarize naïve CD4^+^ T cells towards a Th1/Th17 phenotype, causing immune-mediated injury. Therefore, YY1 may represent a therapeutic target for preventing liver allograft rejection. Future studies should focus on elucidating the molecular mechanisms of YY1’s actions and identifying upstream regulators to further explore its role in transplantation immunology.

## Data Availability

The raw data supporting the conclusions of this article will be made available by the authors, without undue reservation.
